# A randomized comparative study of serial membrane sweeping at term for vaginal birth after caesarean section in two tertiary hospitals in Delta State

**DOI:** 10.11604/pamj.2024.48.60.37918

**Published:** 2024-06-14

**Authors:** Innocent Okoacha, Patrick Ifeanyi Okonta, Osamudia Okhionkpamwonyi

**Affiliations:** 1 Department of Obstetrics and Gynaecology, Delta State University Teaching Hospital, Oghara, Delta State, Nigeria; 2 Department of Obstetrics and Gynaecology, Delta State University, Abraka, Nigeria

**Keywords:** Labour, serial membranes sweeping, vaginal birth after caesarean section (VBAC), Tertiary Hospitals, Nigeria

## Abstract

**Introduction:**

postdate pregnant women with one previous caesarean section that are planned for vaginal birth after caesarean sections are faced with adverse pregnancy outcomes. This trial was conducted to determine the effect of serial membrane sweeping from 38 weeks gestation in pregnant women planned for vaginal birth after caesarean section.

**Methods:**

this randomized controlled trial (RCT) was conducted on 90 women at 38 weeks with one previous caesarean section. In the study group, membranes sweeping commenced at 38 weeks and repeated weekly till labour onset. If no labour onset at 41 weeks and 3 days, elective caesarean section was done. In the control group, patients awaited labour onset till 41 weeks and 3 days, after which elective caesarean section was done. Data collected were analyzed using the Statistical Package for Social Sciences (SPSS ver. 22). All analyses were done at p<0.05.

**Results:**

labour onset before 41 weeks and 3 days was statistically significantly higher in the study group compared to the control group (RR= 1.5; 95% CI: 1.1 - 2.0; P=0.006). Likewise, successful vaginal birth after caesarean section was statistically significantly higher in the study group (RR=1.7; 95% CI: 1.2-2.5; P = 0.001).

**Conclusion:**

serial membrane sweeping from 38 weeks gestation has significant beneficial effect on labour onset and successful vaginal delivery in women with one previous caesarean section.

## Introduction

Pregnant women with one previous caesarean section that are planned for vaginal birth after caesarean section (VBAC) but are postdated constitute a high-risk group in obstetrics. This is due to the likelihood of prolonged pregnancy with its complications, and risk of repeat caesarean delivery with its economic and reproductive implications. Vaginal birth after caesarean section is an important obstetric concept because the caesarean section rates are on the increase worldwide [1], particularly in Nigeria, where incidence of caesarean section (CS) ranges from 20.8 to 34.5% [2-4]. It is an option of delivery that allows women to attempt vaginal delivery after previous CS, and it is clinically safe in carefully selected women [5]. Although it has been reported that the success rate of VBAC ranges from 46 to 75% [6-9], however, failure rate significantly increased in women attempting VBAC at 40 or more weeks of gestation [10,11]. The practice of VBAC is relevant in Nigeria because most pregnant women strongly dislike CS [12,13]. Successful VBAC would reduce the CS rate and complications associated with multiple caesarean deliveries [14,15]. In developing countries, most obstetricians recommend elective repeat CS for postdate pregnancies in women with one previous CS.

This would increase the CS rate and subject the patient to elective CS in subsequent pregnancies, with its attendant complications. Therefore, to reduce the incidence of postdate pregnancies and the associated repeat CS, a simple and efficient technique, that can increase the rate of labour onset and successful VBAC at term, is imperative. Membranes sweeping offers some promises in this regard, as it has been shown to increase labour onset and reduce prolonged pregnancy in low risk pregnancies [16-21]. The effect of routine membrane sweeping in early term in women planned for VBAC remains speculative. Membrane sweeping is a non-pharmacological approach of initiating labour. It causes a rise in the activities of phospholipase A2, prostaglandin F2α, platelet-activating factor, cytokines, and mechanical dilatation of the cervix which releases prostaglandins that facilitate onset of labour [22]. The membranes are swept by inserting the finger into the internal cervical os, and the inferior pole of the membranes is detached from the lower uterine segment [16]. Membrane sweeping improves the favorability of the cervix; initiates spontaneous labour and reduces the number of prolong pregnancies [19,23]. Side effects like pain and vaginal bleeding may be present [23,24].

Only few studies have been done in affluent nations on the impact of membrane sweeping in women with one prior CS, and the results of such studies were conflictive [25-27]. The effectiveness of membrane sweeping on labour onset and successful VBAC before 41 weeks was demonstrated by Afzal *et al*. [25] in comparison to patients who did not have membrane sweeping. On the contrary, Hamdan *et al*. [27] concluded that serial membrane sweeping at term has no appreciable impact on the start of labour, pregnancy duration, or repeat caesarean delivery. To the best of our knowledge, study on the impact of serial membrane sweeping at term in women with one prior lower segment CS has not been done in Nigeria, and conducting the study at the Delta State University Teaching Hospital (DELSUTH), Oghara, and Central Hospital, Warri, Delta State, would add to the existing knowledge from the developed countries. It will further provide evidence to either support or refute the practice of serial membrane sweeping in women planned for VBAC.

## Methods

**Study design:** this was a randomized control study with two groups of participants: in the intervention group (membranes sweeping), the participants had fetal membranes separated from the lower uterine segment, while in the control group (no membranes sweeping group), fetal membranes was not separated from the lower uterine segment. Participants were equally allocated to the study groups.

**Study setting:** this study was conducted at the department of Obstetrics and Gynaecology, DELSUTH, Oghara, and Central Hospital, Warri, Delta State. The two hospitals have similar clinical management protocols and provide specialist care to patients. The combined average pregnancy delivery rate was 5220 per annum.

**Study population:** this consisted of pregnant women from 38 weeks gestational age with one prior CS, who were planned for VBAC. The randomized controlled trial (RCT) was conducted between April and October 2018.

**Inclusion criteria:** these included women with one previous CS with non-recurrent indications, singleton pregnancy with foetus in cephalic presentation at 38 weeks´ gestation, intact membranes, participants willing to undertake VBAC and gave consent to participate in the study.

**Exclusion criteria:** these included multiple gestations, malpresentations, placenta praevia, abruption placentae, cephalo-pelvic disproportion, fetal macrosomia, obstructive pelvic masses, congenital anomalies and maternal medical disorders.

**Sample size calculation:** the sample size per group was determined using the formula for sample size calculation for clinical intervention comparative studies with qualitative endpoint [28].


$$N=\frac{2{\left(z_{\alpha }+z_{\beta }\right)}^{2}p\left(1-p\right)}{{\left(P_{1}-P_{1}\right)}^{2}}$$

Zα = standard normal variate at 5% level of significance (p-value 0.05) = 1.96; Zβ = standard normal variate at 80% power = 0.84; P_2_ = proportion with successful VBAC “membranes sweeping group”= 61.82% +27.27% =89.09% [25] = 0.8909; P_1_ = proportion with successful VBAC “no membranes sweeping group” =25.45+32.73 =58.18 [25] = 0.5818; P = pooled prevalence = (0.5818 + 0.8909)/2= 0.73635.


$$N=\frac{2{\left(1.96+0.84\right)}^{2}0.7364\left(1-0.7364\right)}{{\left(0.5818-0.8909\right)}^{2}}=32$$

To accommodate a 10% loss to follow-up, a minimum of 35 patients per arm were required for this study.

**Randomization:** patients were randomly allocated into one of the two study groups, using numerically ordered cards in sealed envelopes. Ninety 4cm x 4cm blue cards were numbered 01 to 90, and each was sealed in identical opaque envelopes. The envelopes were placed in a safe cupboard in the antenatal clinic of the two centres from there they were drawn serially until the study was completed. The participants with even and odd numbers were allocated to membrane sweeping and no membrane sweeping groups, respectively. The group to which the patient was allocated was only known after the envelope was opened.

**Study interventions:** two research assistants (senior registrars) were trained for the study. All patients recruited at DELSUTH were examined and data collected by the principal investigator, while all patients recruited at the Central Hospital were examined and data collected by a trained senior registrar. Weekly follow-up at the antenatal clinic with the investigators were arranged until delivery. Participants allocated to the intervention group had their membranes swept at the labour ward of the hospitals. With the woman in dorsal position, initial cervical assessment for the Bishop Score was done. Thereafter, the investigator´s examining finger was introduced into the cervical os and the fetal membranes were digitally separated from the lower uterine segment by two circular movements of the examining finger [16]. When digital separation of fetal membranes was not possible because the cervix was closed, massage of the surface of the cervix was done with circular pushing and massaging movements of the examining fingers for approximately 30 seconds [19]. Each participant was observed for 1 hour in the labour after the procedure. Participants assigned to the control group had only vaginal examination to assess the bishop score.

Participants were encouraged to present to labour ward when they experience features of labour. At the labour ward, the gestational age and the time of labour onset were recorded. For this trial, spontaneous labour was defined as self-presentation of a participant into the labour ward with regular painful uterine contractions occurring at least once in 10 minutes. Active management of labour was adopted regardless of the study group, using standard protocol of the hospitals. Cases of premature rupture of membranes (PPROM) were managed using the standard hospital protocols. Successful VBAC was regarded as a vaginal birth in a woman who has a previous CS. For this study, failure of a pregnant woman to achieve spontaneous labour at 41 weeks and 3 days in any group was regarded as ‘prolonged pregnancy’ and necessitated CS.

**Outcome measures:** the primary outcome measure was the proportion of women with successful VBAC. The secondary outcome measures were proportion that achieved spontaneous labour, gestational age at onset of labour, number of membrane sweeping to initiate labour, sweeping and pelvic examination to delivery interval, mode of delivery, prelabour rupture of membrane, vaginal bleeding and fetal outcomes.

**Figure 1:**
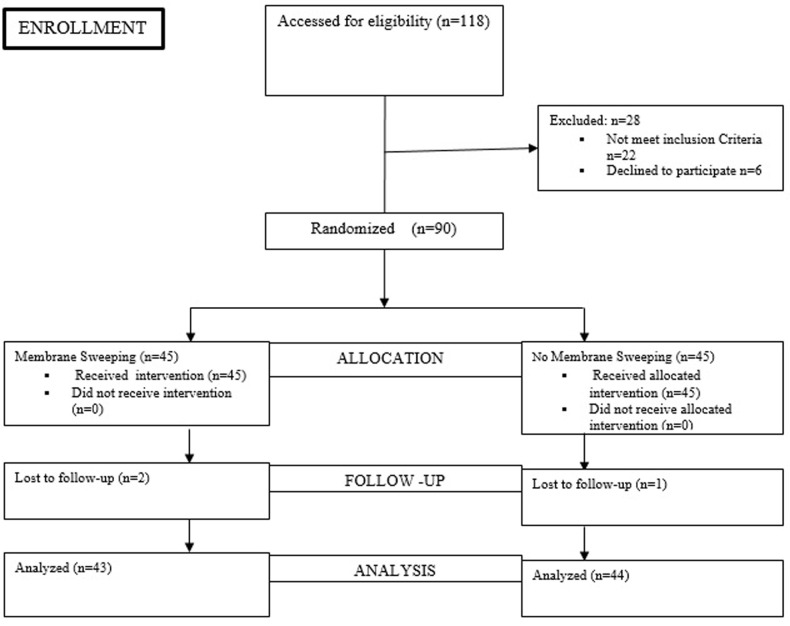
consort algorithm of the randomization and follow-up of study participants

**Data collection and analysis:** data collection was facilitated by the principal investigator and his assistants using a specially designed data collection sheet. Data was analyzed using Statistical Package for Social Sciences version 22 (IBM® Inc, Il Chicago. USA). Comparisons of patients´ characteristics and outcome measures were conducted using the Chi-Square tests (with Fisher´s Exact test when necessary) for categorical variables, and the Student´s t-test for continuous variables. Relationships were expressed using relative risks and confidence intervals. Statistical significance was considered to be at a probability value of < 0.05. A summary of the study from recruitment to data analysis is shown in Figure 1.

## Results

One hundred and eighteen pregnant women with one prior CS were assessed for eligibility. Twenty-eight were excluded because, either, they did not meet the inclusion criteria (n=22) or refused to participate (n=6). Ninety participants were equally randomized to either the membrane sweeping (n=45) or no membrane sweeping group (n=45). Three participants were lost to follow-up because they did not deliver at the study centres. The base-line demographic, clinical characteristics and anthropometric indices were similar in both groups (Table 1). In Table 2 showed that the proportion of the participants who had successful VBAC was statistically significantly higher in membrane sweeping compared to the no membrane sweeping group (34/43 {79.1 %} vs. 20/44 {45.5 %}; RR=1.7; 95% CI: 1.2-2.5; P = 0.001). In Table 3 shows the secondary outcome measures of the study. The proportion of patients that had labour onset before 41 weeks 3 days gestation was significantly higher in the membrane sweeping than the no membrane sweeping group (36/43 {83.7%} vs. 25/44 {56.8%}; RR= 1.5; 95% CI: 1.1 - 2.0; P=0.006), respectively (Table 3).

**Table 1:** baseline demographic, clinical characteristics and anthropometric indices

Variables	Categories	Study groups		Test statistics	P -value
		Membrane sweeping	No membrane sweeping		
Age (years)	15 – 24 years	6 (14.0)	4 (4.1)	χ2=0.652	0.884
	25 – 34 years	24 (55.6)	25 (56.8)		
	35 – 44 years	11 (25.6)	12 (27.3)		
	>45 years	2 (4.7)	3 (6.8)		
	Mean ± SD	31.5 ± 6.4	31.6 ± 6.0	t= 0.060	0.952
Parity	Primipara	7 (16.3)	11 (25.0)	χ2=1.893	0.388
	Multipara	33 (76.7)	32 (72.7)		
	Grand multipara	3 (7.0)	1 (2.3)		
	Median	2.0	3.0		*0.585
Previous VBAC	Present	13 (30.2)	12 (27.3)	χ2=0.0093	0.760
	Absent	30 (69.8)	32 (72.7)		
		RR:1.2 (0.46-2.9)			
Previous vaginal delivery	Present	36 (83.7)	34 (77.3)	χ2=0.575	0.448
	Absent	7 (16.3)	10 (22.7)		
		RR: 1.5(0.4 – 4.4)			
G.A at recruitment	Mean ± SD	38.4 ± 0.2	38.3 ± 0.2	t= 0.038	0.970
Bishop score at recruitment	0 - 5	31 (72.1)	34 (77.3)	χ2=0.309	0.578
	6 - 13	12 (27.9)	10 (22.7)		
		RR: 0.76(0.291.98)			
	Mean ± SD	4.2 ± 2.03	4.3 ± 1.68	t= 0.101	0.057
BMI (kg/m^2^)	Normal	2 (4.7)	5 (11.4)	χ2=1.774	0.412
	Overweight	36 (83.7)	36 (81.8)		
	Obese	5 (11.6)	3 (6.8)		
	Mean ± SD	27.3 ± 2.3	26.7 ± 2.4	t = 1.328	0.188
Weight (kg)	Mean ± SD	70.1 ± 7.1	68.6 ± 6.2	t = 1.048	0.298
Height (m)	Mean ± SD	1.6 ± 0.1	1.6 ± 0.1	t = 0.305	0.761

t: student t-test for two independent means; * Fishers exact; χ2: chi-squared; BMI: body mass index; RR: relative risk; VBAC: vaginal birth after caesarean section; G.A: gestational age

**Table 2:** successful vaginal birth after caesarean section (VBAC) among participants

Variables	Categories	Successful VBAC		χ2	p-value
		Present	Absent		
Study group	Membrane sweeping	34 (79.1)	9 (20.9)	10.438	**0.001**
	No membrane sweeping	20 (45.5)	24 (54.5)		
		RR: 1.7; 95% CI:1.2-2.5			

CI: confidence interval; RR: relative risk; numbers in bold: statistically significant

**Table 3:** secondary outcome measures among participants

Variables	Categories	Study groups		Test statistics	p-value
		Membrane sweeping	No membrane sweeping		
Spontaneous labour	Present	36 (83.7)	25 (56.8)	χ2=7.512	*0.006
	Absent	7 (16.3)	19 (43.2)		
		RR: 1.5; 95% C1: 1.1 – 2.0			
GA at labour onset	Mean ± SD	39.2 ± 0.8	39.7 ± 0.7	χ2=2.411	*0.019
PROM	Present	7 (16.3)	5 (11.4)	χ2=0.442	0.506
	Absent	36 (83.7)	39 (88.6)		
		RR: 1.4; 95% CI: 0.5 – 4.2			
Vaginal bleeding	Present	1 (2.3)	0 (0.0)	χ2=1.035	0.494
	Absent	42 (97.7)	44 (100.0)		
GA at delivery	Mean ± SD	39.5 ± 1.0	40.2 ± 0.9	t= 3.142	*0.002
Recruitment to delivery Interval (days)	1-3	8 (18.6)	1 (2.3)	χ2=10.232	*0.006
	4-10	21 (48.8)	16 (36.4)		
	>10	14 (32.4)	27 (51.3)		
	Mean ± SD	8.8 ± 6.5	12.9 ± 5.5	t= 3.193	*0.002
Mode of delivery	VBAC	34 (79.1)	20 (45.5)	χ2=10.438	*0.001
	Repeat CS	9 (20.9)	24 (54.5)		
		RR: 1.7; 95% CI:1.2 – 2.5			

CS: caesarean section; VBAC: vaginal birth after caesarian section; t: Student t-test; χ2: Chi square; RR: relative risk; numbers in bold: statistically significant; PROM: premature rupture of membranes

**Table 4:** neonatal outcomes among participants

Variables	Categories	Study groups		Test statistics	P-value
		Membrane sweeping	No membrane sweeping		
First-minute APGAR score	<7	4 (9.3)	2 (4.5)	χ2=0.736	0.434
	≥7	39 (90.7)	42 (95.5)		
		RR: 1.0; 95% CI: 0.9 – 1.1			
Five minutes APGAR Score	<7	1 (2.3)	2 (4.5)	*0.322	1.000
	≥7	42 (97.7)	42 (95.5)		
		RR: 1.0; 95% CI: 1.0 – 1.1			
Birth weight	<2.5 kg	1 (2.3)	2 (4.5)	*0.679	1.000
	2.5 - 4.0 kg	40 (93.0)	39 (88.6)		
	>4.0 kg	2 (4.7)	3 (6.8)		
	Mean ± SD	3.3 ± 0.5	3.2 ± 0.5	t= 0.697	0.488
NICU admission	Yes	1 (2.3)	2 (4.5)	*0.322	1.000
	No	42 (97.7)	42 (95.5)		
		RR: 0.5; 95% CI: 0.04 – 5.6			

NICU: Neonatal Intensive Care Unit; t: student t-test; *Fishers exact

**Figure 2:**
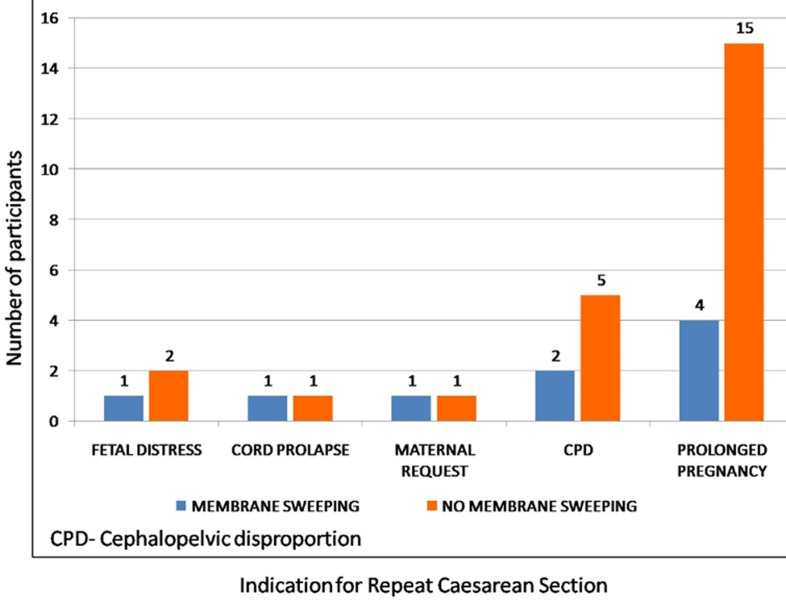
indications for repeat caesarean section

**Figure 3:**
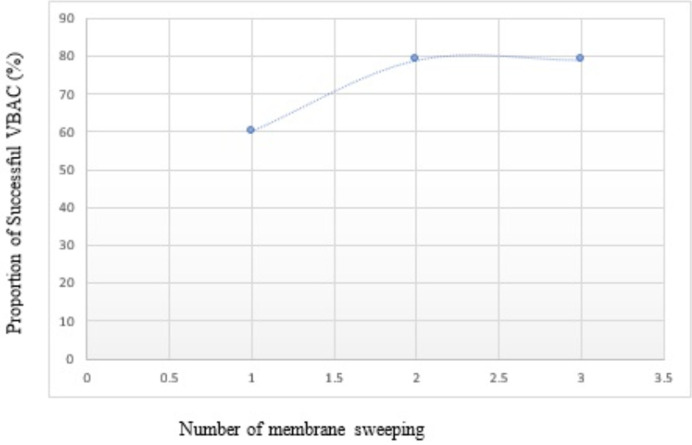
proportion of successful vaginal birth after caesarean section (VBAC) with number of membrane sweeping

The mean gestational ages at onset of labour for the study and control groups were 39.2 ± 0.8 and 39.7 ± 0.7, respectively. The difference was statistically significant (P= 0.019). Correspondingly, the mean gestational age at delivery was significantly lower in membrane sweeping compared to no membrane sweeping group (39.5 ± 1.0 vs. 40.2 ± 0.9; P=0.002). Membrane's sweeping reduced the mean recruitment to delivery interval by 4 days (8.8 ± 6.5 vs. 12.9 ± 5.5; P=0.002). More participants had repeat CS in the no membrane sweeping compared to the membrane sweeping group, with a statistically significant difference (24/44 {54.5%} vs. 9/43 {20.9%}; RR=1.7; 95% CI: 1.2 - 2.5; P=0.001). More participants in the membrane sweeping group had PROM, the difference was not statistically significant (7/43 {16.3%} vs. 5/44 {11.4%}; RR= 1.4; 95% CI: 0.5- 4.17; P=0.506). Only one participant in the membrane sweeping group complained of vaginal bleeding during the study period. A greater proportion of participants in the no membrane sweeping group had repeat CS due to prolonged pregnancy (Figure 2). However, there was no significant difference in the various indications for repeat CS between the groups (P=0.690). There was 60% successful VBAC after the first membrane sweeping among participants; successful VBAC increased to 79% after the second membrane sweeping; and successful VBAC rate remained 79% after the third membrane sweeping, as shown in Figure 3.

## Discussion

We conducted a randomized controlled trial to assess the impact of membranes sweeping from 38 weeks gestation in women with one previous CS planned for VBAC. The base-line socio-demographic features, clinical characteristics and anthropometric indices are similar in the two groups. This shows that the randomization is able to eliminate selection bias that has the potential of undermining the validity of the results in our study. Our study shows 79.1% successful VBAC after membrane sweeping in patients with one previous CS, which is comparable with a study conducted by Afzal *et al*. which showed that 61.8% of patients had successful VBAC after membranes sweeping [25]. Although the study by Afzal *et al*. has a lower proportion of women with vaginal delivery, however, both studies show that the percentage of the participants that had vaginal delivery were significantly higher in membrane sweeping compared to the no membrane sweeping group [25]. Our findings of 79.1% and 45.5% of successful VBAC in the study and control groups, respectively, are higher than the successful VBAC rate of 17.3% for the membrane sweeping group and 18.7% for the no membrane sweeping group reported by Ramya *et al*. [26]. Similarly, the proportion of women that had successful VBAC in our study was higher than the 59.8% reported by Hamdan *et al*. [27]. Hamidi and colleagues in a systematic review and meta-analysis of two RCT were unable to decide whether membrane sweeping was a beneficial way of achieving spontaneous labour and vaginal delivery in patients undergoing planned VBAC [29].

The statistically significant increase in successful VBAC after membrane sweeping in our study (34/43 {79.1%} vs. 20/44 {45.5 %}; RR=1.7; 95% CI: 1.2-2.5; P = 0.001) could be due to good patient selection, proportion of participants with prior vaginal delivery, higher mean gestational age at recruitment, higher mean Bishops score at recruitment (BS=4.23 ± 2.03) and careful monitoring of the patients in labour with partograph. It is most likely that this subset of term pregnant women have more favourable cervical status and thus, eventual more efficient membranes sweeping, since the effect of membrane sweeping increases with gestational age and Bishop score [21]. In this study, 83.7% of participants in the membrane sweeping group had labour onset at a mean gestational age of 39.2 ± 0.8 and 56.8% of patients in the no membrane sweeping group achieved labour onset at a mean gestational age of 39.7 ± 0.7. The difference is statistically significant. This is similar to a trial by Afzal *et al*. [25], which showed that 78.2% of patients in the membrane sweeping group had onset of labour, which was significantly higher than the 50.1% of participants with onset of labour in the control group. Our finding is in contrast to the trial by Ramya *et al*. [26,27], which reported no significant difference in the onsets of labour and the gestational ages at onset of labour.

This may have been due to the reported high rate of opting out of the patients from their study in favour of repeat CS on maternal request before 41 weeks of gestation, which probably reduced the number of participants that would have achieved successful VBAC. Also, Ramya *et al*. and Hamda *et al*. respectively, reported very low mean Bishop´s scores of 1 and 2 in the membrane sweeping group at recruitment, hence the cervix may not be adequately dilated for the examining finger(s) to separate the fetal membranes from the lower uterine segment [26,27]. The mean gestational age at delivery is significantly lower in membrane sweeping compared to no membrane sweeping group (P=0.002). This is in contrast with findings in the studies conducted by Ramya *et al*. and Hamdan *et al*. which did not show any significant difference in the mean gestational ages [26,27]. There is a 60% successful VBAC after the first membrane sweeping among participants. The proportion of participants with successful VBAC increases to 79% after the second membrane sweeping. After the third membrane sweeping, the successful VBAC rate remains at 79%. Also, membranes sweeping reduces the mean recruitment to delivery interval by 4 days (8.8 ± 6.5 vs. 12.9 ± 5.5; P=0.002). This is probably because the weekly membranes sweeping in our study puts patients in a prelabour state in which irregular uterine contractions have a cervical ripening effect, and improves the bishop score, with labour onset and eventual vaginal delivery at earlier gestational age.

The proportion of participants with repeat CS is more in the no membrane sweeping compared to the membrane sweeping group, and the difference is statistically significant (P=0.001). Although previous studies revealed higher rate of repeat CS than our study, they did not show any significant difference in the rate of repeat CS between participants in both groups. The high rate of repeat CS was due to the maternal request after the onset of labour [26,27,29]. There is no significant maternal or fetal complication in our study. The women who received membranes sweeping have more incidence of prelabour rupture of membranes, but the difference is not statistically significant (P=0.5). This finding is in agreement with studies in women with low-risk pregnancies [19,21]. More participants in the no membrane sweeping group had repeat CS due to prolonged pregnancy. However, there was no significant difference in the various indications for repeat CS between the groups (P=0.7). The result is similar to the trial by Ramya *et al*. [26], Hamdan *et al*. [27]. Likewise, the neonatal outcomes in terms of the appearance pulse, grimace, activity and respiration (APGAR) scores, birth weight and need for admission into the neonatal intensive care unit (NICU) were similar in both groups. All these findings are in consonance with previous studies on membrane sweeping at term [21,27].

The strength of our study was the randomized allocation of participants to the study groups, which minimized selection bias and unequal allocation of confounders among the participants. Schultz *et al*. [30] described randomized allocation of participants as the most effective means of minimizing unequal allocation of potential confounders among participants of a clinical trial. Each of the two centres of our study had one trained research assistant, in addition to the principal investigator, and they performed the cervical assessment for Bishop scoring to reduce variability. The combination of two centers for the study guaranteed availability of large numbers of potential participants. Additionally, the trial was on a group of high-risk patients that have not been extensively studied.

**Limitations**: this study is not without limitations. The investigator and research assistants who were involved in data collection were not blinded to the allocation arms. This was not so much a problem because the outcome measures were fairly objective and not assessors´ dependent. Our participants comprised of women with prior vaginal birth as well as women without vaginal birth who were at increased risk of repeat CS. With the established favorable outcome of planned VBAC in women with prior vaginal birth, inclusion of these subsets of women may have further reduced the power of our study.

## Conclusion

This RCT shows statistically significant difference in onset of labour at term, gestational age at onset of labour, recruitment to delivery interval, gestational age at delivery, successful VBAC, and repeat CS rate in patients who had serial membrane sweeping compared with patients who had no membrane sweeping. Furthermore, there is no significant detrimental effect of membranes sweeping to the patients. Serial membrane sweeping at term can, therefore, be used routinely to increase the rate of successful VBAC in women with one previous CS. We also recommend more studies on this subject.

### What is known about this topic

- *Sweeping of membranes is a simple non-pharmacological way of inducing labour;*
- *Sweeping of membranes promotes the onset of labour at term in previous one caesarean section in comparison with no membrane sweeping;*
- *Few studies have been done with contradictory findings*.

### What this study adds

- *Serial membrane sweeping from 38 weeks gestation has a significant beneficial effect on labour onset and successful vaginal delivery in women with one previous caesarean section;*
- *There was a 60% successful VBAC after the first membrane sweeping, which increased to 79% after the second membrane sweeping but remained at 79% following the third membrane sweeping;*
- *Membrane sweeping reduces the mean recruitment to delivery interval by 4 days*.
